# Supplementary material for: A weakened interface in the P182L variant of HSP27 associated with severe Charcot‐Marie‐Tooth neuropathy causes aberrant binding to interacting proteins
Source: EMBO J. 2021 Mar 1;40(8):e103811. doi: 10.15252/embj.2019103811 (PMC8047445; doi:10.15252/embj.2019103811)

**Appendix**

**The neuropathy-causing P182L mutation dysregulates interactions of HSP27**

T. Reid Alderson^§^, Elias Adriaenssens^§^, Bob Asselbergh, Iva Pritišanac, Jonas Van Lent, Heidi Y. Gastall, Marielle A. Wälti, John M. Louis,

Vincent Timmerman^*^, Andrew J. Baldwin^*^, Justin L. P. Benesch^*^

* Corresponding authors

^§^ These authors contributed equally

**Table of Contents**

1. **Appendix Figure S1**: Control experiments for P182L and HSP27 self-aggregation

2. **Appendix Figure S2**: Negative-stain electron microscopy of HSP27

3. **Appendix Figure S3**: The P182L mutation increases the number of cells with large cytoplasmic insoluble aggregates under similar HSP27 expression levels.

4. **Appendix Figure S4**: Hetero-oligomerization between WT and P182L HSP27

5-6. **Appendix Figure S5**: Analysis of the peptide binding NMR titration data with the TITAN software.

7-8. **Appendix Figure S6**: Resonances in the β4/β8 groove disappear upon peptide binding and only reappear at higher temperatures

9 **Appendix Figure S7**: Recovery of NMR signal intensities in peptide-bound cHSP27 at high temperature

10. **Appendix Figure S8**: ^15^N CPMG relaxation dispersion data for peptide-bound cHSP27

11. **Appendix Figure S9**: Concentration-dependent ^15^N dispersions for peptide-bound cHSP27

12. **Appendix Figure S10**: cHSP27 adopts similar conformations upon binding WT or P182L peptides.

13. **Appendix Figure S11**: Global analysis of the dispersion and titration data

14. **Appendix Figure S12**: Bioinformatics analyses of [V/I]x[V/I] motifs in the human proteome.

15. **Appendix Figure S13**: Co-immunoprecipitation of BAG3 by WT HSP27

16. **Appendix Figure S14**: Interactions between the non-IxI/V residues in the peptide and the ACD

17. **Appendix Table S1**: ^15^N |Δω| values for cHSP27 sparsely bound to WT or P182L peptides.

18. **Appendix Table S2**: Kinetic parameters for WT and P182L peptide binding to cHSP27

19. **Appendix Table S3**: Energetic parameters for WT and P182L peptide binding to cHSP27

20. **Appendix Table S3**: Proteome-wide analysis of IxI/V motifs.

21-22. **Appendix Table S4**: Known HSP27-interacting proteins that also contain [I/V]P[I/V] motifs.

23. **Table of Contents Graphic**

**Appendix Figure S1**


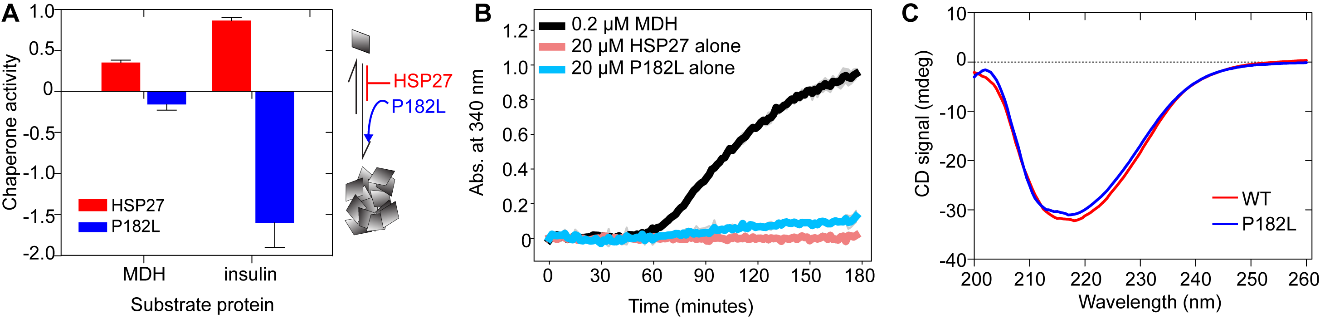


**Appendix Figure S1.** **Control experiments for P182L and HSP27 self-aggregation.** (**A**) The chaperone activity of HSP27 (red) and the P182L variant (blue) against MDH and insulin. Chaperone activity is defined as 1 – ϴ, where ϴ is the maximum intensity for the chaperone plus substrate mixture divided by the maximum intensity of the substrate alone. Values of 1 and 0 respectively indicate maximum protection and the absence of protein against aggregation. (**B**) Data from Figure 2 depicting the aggregation of 0.2 µM malate dehydrogenase (MDH, black) at 40 °C in the presence and absence of 0.5 µM HSP27 (red) or 0.5 µM P182L (blue). Alongside these experiments, controls were performed in which 20 µM of HSP27 (light red) and 20 µM of P182L (light blue) were incubated at 40 °C. There is no absorbance at 340 nm (Abs. 340 nm) for 20 µM HSP27, indicating the absence of aggregation, and only minor signals for 20 µM P182L. The buffer conditions were 30 mM sodium phosphate, 100 mM NaCl, 2 mM EDTA at pH 7. (**C**) Circular dichroism (CD) spectra of WT HSP27 (red) and the P182L variant (blue). Both samples were prepared in 20 mM sodium phosphate, 100 mM NaCl, 2 mM EDTA at pH 7.4. The total protein concentration was 20 µM for both samples, and the spectra were collected at 20 °C.

**Appendix Figure S2**


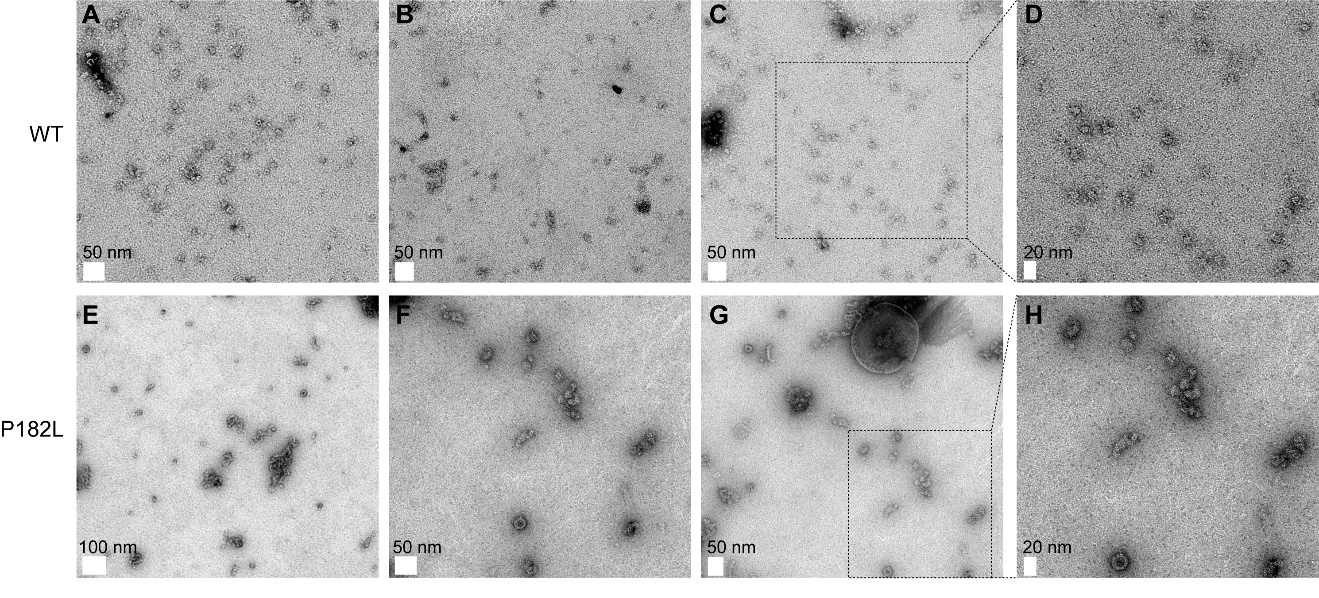


**Appendix Figure S2.** **Negative-stain electron microscopy of HSP27.** Negative-stain electron microscopy (EM) images for WT (**A-D**) and P182L (**E-H**). Scale bars are depicted in the lower left corner of each image. The dashed boxes in panels C and G correspond to the regions that were subsequently imaged at higher magnification in panels D and H, respectively.

**Appendix Figure S3**

**
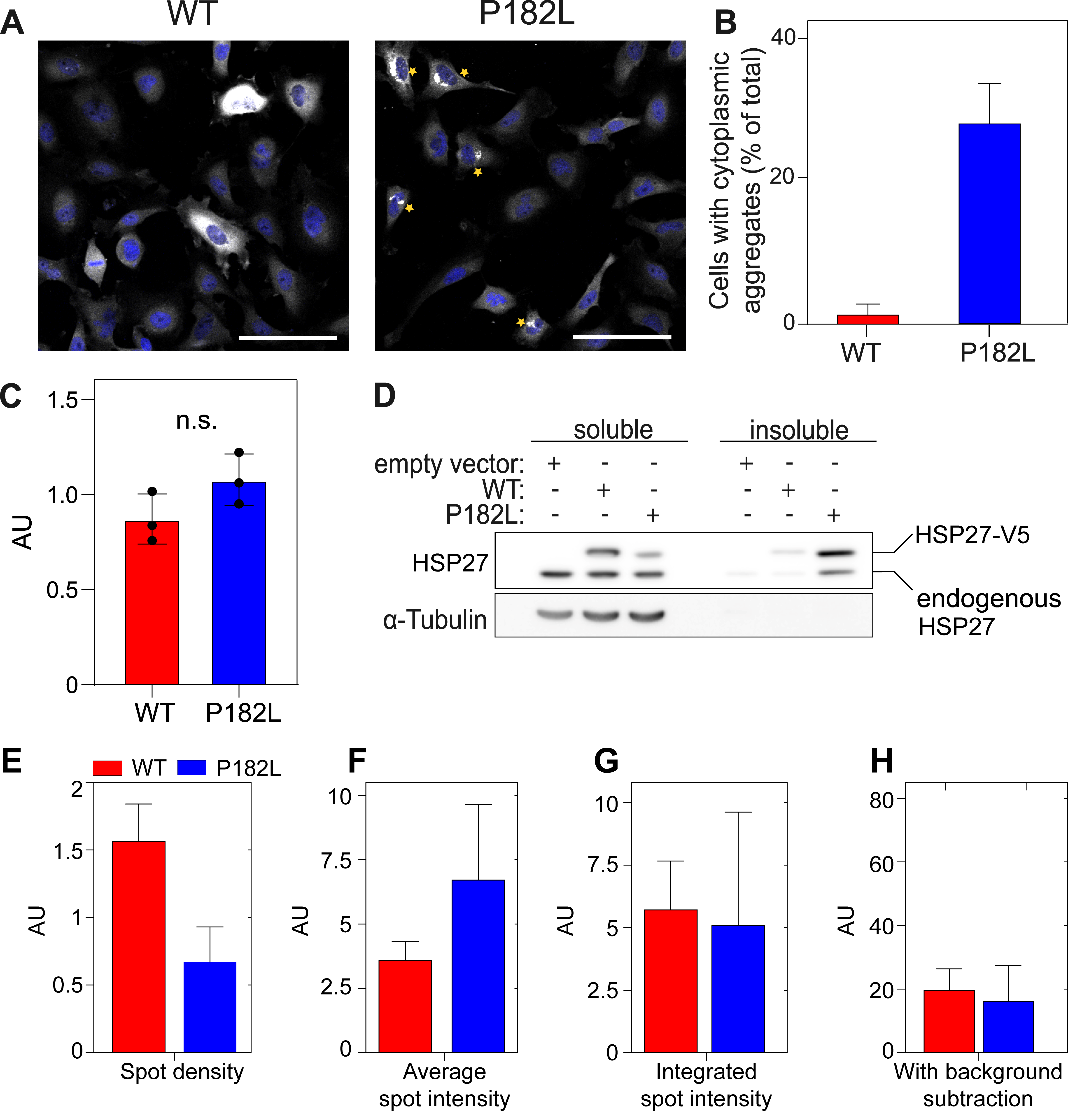
**

**Appendix Figure S3.** **The P182L mutation increases the number of cells with large cytoplasmic insoluble aggregates under similar HSP27 expression levels.** (**A**) Expression of V5-epitope-tagged HSP27 (WT or P182L mutant) in HSP27 knock-out HeLa cells and immunostaining of HSP27. Scale bar = 100µm. Asterisks indicate cells with aggregates. (**B**) All cells present in images of random large microscopic fields (640 µm x 640 µm) were visually scored for the presence of large high intensity aggregates. In total, 1361 cells were evaluated in 14 microscopic fields. The bar graph showing the percentage of total cells with cytoplasmic aggregates. The error bar represents the standard deviation between the different microscopic fields. (**C**) Quantification of the expression level of WT or P182L HSP27. The total level is calculated by summing up the amount of protein in the soluble and insoluble fractions in three independent experiments. The samples were obtained from HeLa cells transiently expressing V5 epitope-tagged HSP27 or the P182L variant. (**D**) A representative western blot from the quantification in panel C and underlying the data in panels E-H. The increased insolubilization of endogenous WT HSP27 in the presence of P182L can be observed in the P182L lane of the insoluble fraction. (**E-H**) Extracting HSP27 expression directly from fluorescence intensities of expansion microscopy images reflects similar expression levels for WT HSP27 and P182L. Multiplying the average spot intensity (**E**) with the number of spots per area, or density, (**F**) yields the same integrated spot intensity (**G**) for both genotypes. The same data were used to plot Figure 2D and E. (**H**) Measurement of mean intensities on the entire image regions confirms similar HSP27 expression in the examined samples

**Appendix Figure S4**


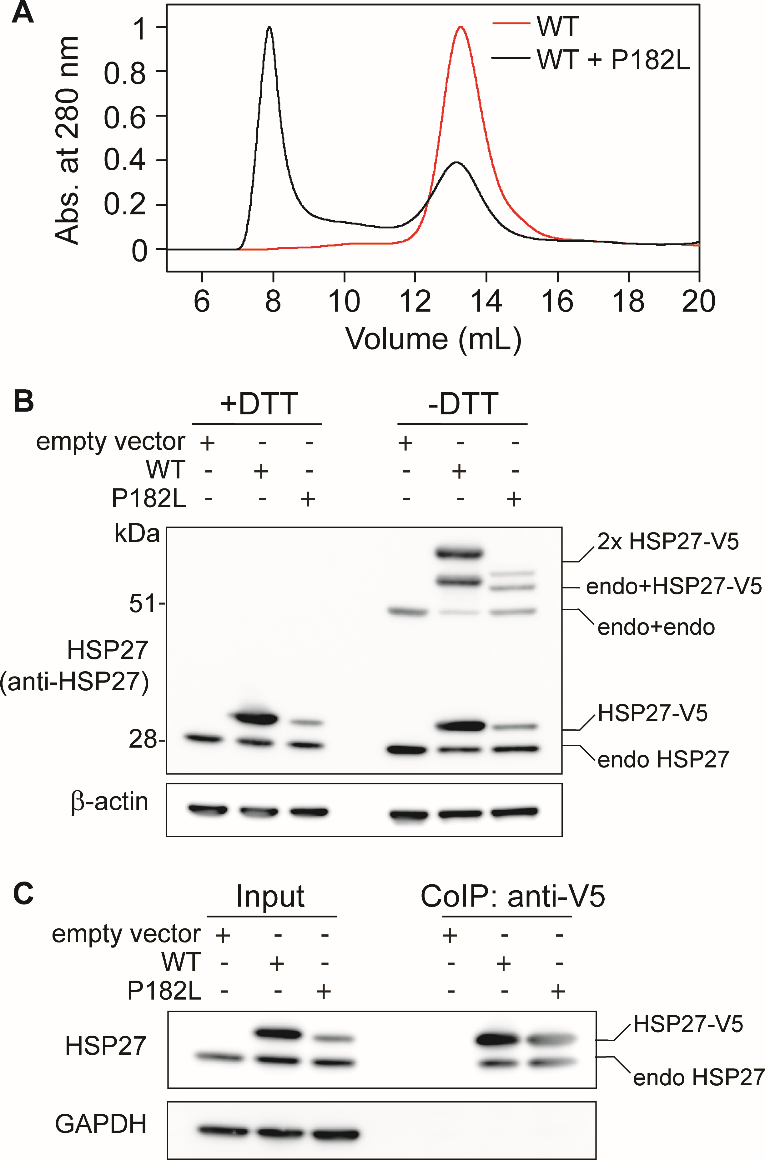


**Appendix Figure S4.** **Hetero-oligomerization between WT HSP27 and the P182L variant in cells and *in vitro*.** (**A**) Equimolar ratios of WT and P182L HSP27 (230 µg each) were mixed and equilibrated overnight at room temperature in 20 mM sodium phosphate, 100 mM NaCl, 2 mM EDTA at pH 7. The sample was then injected onto a Superose 6 10/300 column (black). The same amount of WT HSP27 (230 µg) was injected onto the column (red). The depletion of the WT HSP27 elution peak at *ca.* 13.8 mL in the mixture indicates the formation of hetero-oligomers between P182L and WT HSP27. (**B**) Western blot showing HSP27 monomers (+DTT) and dimers (-DTT). The same protein lysates were prepared in sample buffer with or without DTT. The absence of DTT allowed the visualization of dimers formed through a disulfide bond involving C137, the lone cysteine residue in HSP27. On the right: endo stands for endogenous HSP27 (WT) and 2x stands for a covalently linked dimer via disulfide-bonded C137. (**C**) Co-immunoprecipitation from HeLa cells stably overexpressing V5-epitope-tagged HSP27 (WT or P182L mutant). Shown here is a western blot for anti-V5. The presence of endogenous WT HSP27 (endo) is observed both the WT-V5 and P182L-V5 samples, indicative of hetero-oligomerization.

**Appendix Figure S5**


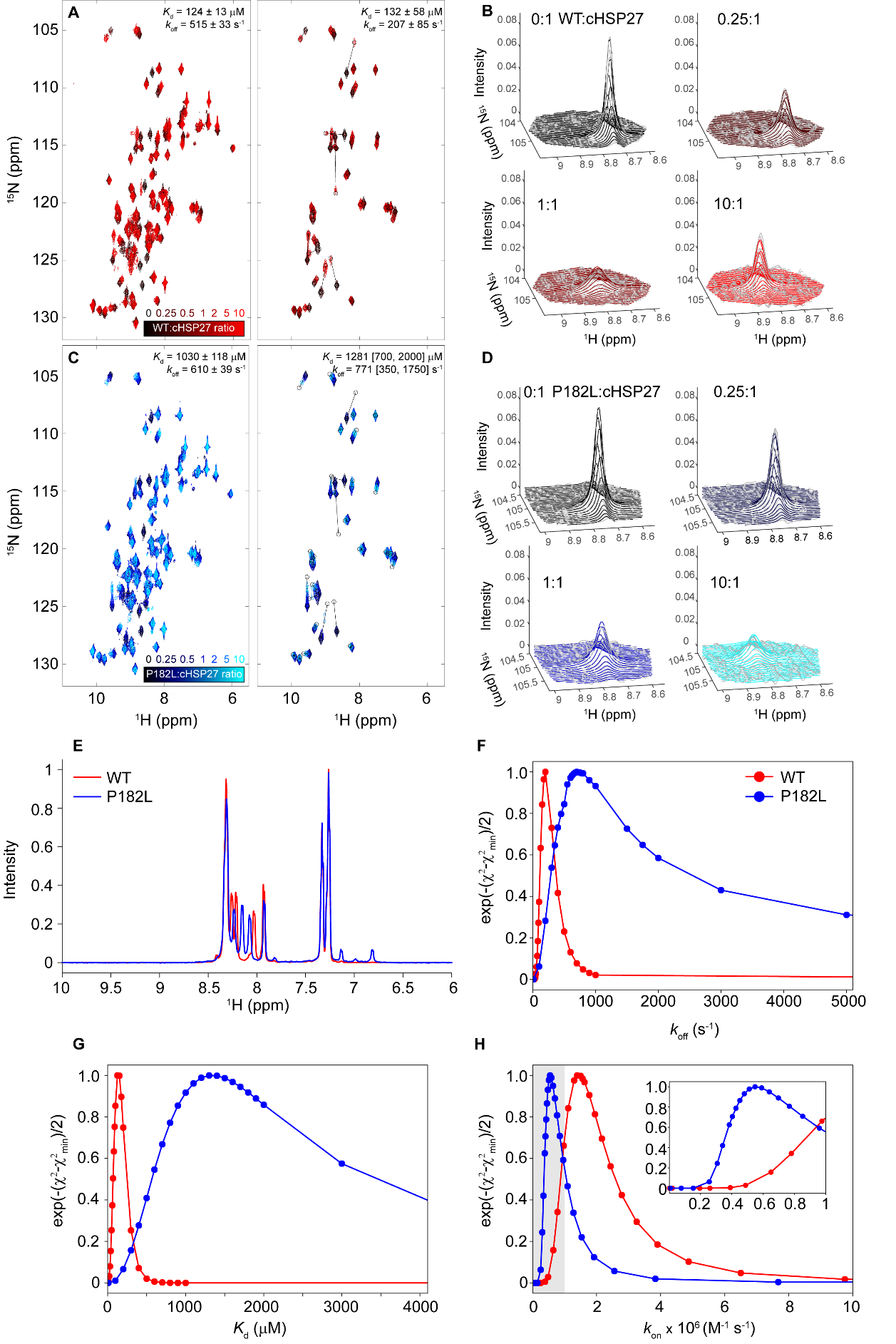


**Appendix Figure S5.** **Analysis of the peptide binding NMR titration data with the TITAN software.** (**A**) *left:* Experimental 2D ^1^H-^15^N HSQC spectra of ^15^N-cHSP27 (black) in the presence of increasing amounts of unlabeled WT peptide (red color gradient). The globally determined *K*_d_ from fitting resonances in fast exchange to equation 1 is listed in the upper-right corner. The *k*_off_ listed is derived from CPMG RD experiments (*vide infra*). *Right*: The software TITAN fits NMR titration data by numerically simulating 2D spectra in the presence of chemical exchange. The *K*_d_ and *k*_off_ fitted by TITAN are listed in the upper-right corner. The color scheme is the same as the left panel. (**B**) For the resonances in panel A, selected peak shapes (grey lines) and the corresponding fits (colored lines) by TITAN are shown. (**C**, **D**) the same as panels A and B except for the corresponding experiments with the P182L peptide. (**E**) Comparison of ^1^H NMR spectra of the wild-type (red) and P182L (blue) peptides. The samples were prepared by dissolving natural abundance peptides at a concentration of 2 mM in 30 mM sodium phosphate, 2 mM EDTA at pH 7 with 6% D_2_O added to maintain lock. The intensities are normalized to the most intense resonance line. (**F**-**H**) TITAN error analyses for *k*_off_ (**F**), *K*_d_ (**G**), and *k*_on_ (**H**) for the titration data of ^15^N-cHSP27 in the presence of the WT peptide (red) or P182L peptide (blue). The χ^2^ value from a fit of the data was tabulated in which either *k*_off_ or *K*_d_ was fixed and the other parameter varied. The probability distribution, exp(-(χ^2^-χ^2^_min_)/2) was then computed and plotted. Because TITAN only fits *k*_off_ and *K*_d_, with *k*_on_ computed from the known relation (*K*_d_ = *k*_off_/*k*_on_), we computed the probability distribution for *k*_on_ by fixing *k*_off_ at its best-fit value while *K*_d_ (*i.e.*, *k*_on_) was iterated. In panel H, the grey region is shown in the inset for clarity.

**Appendix Figure S6**


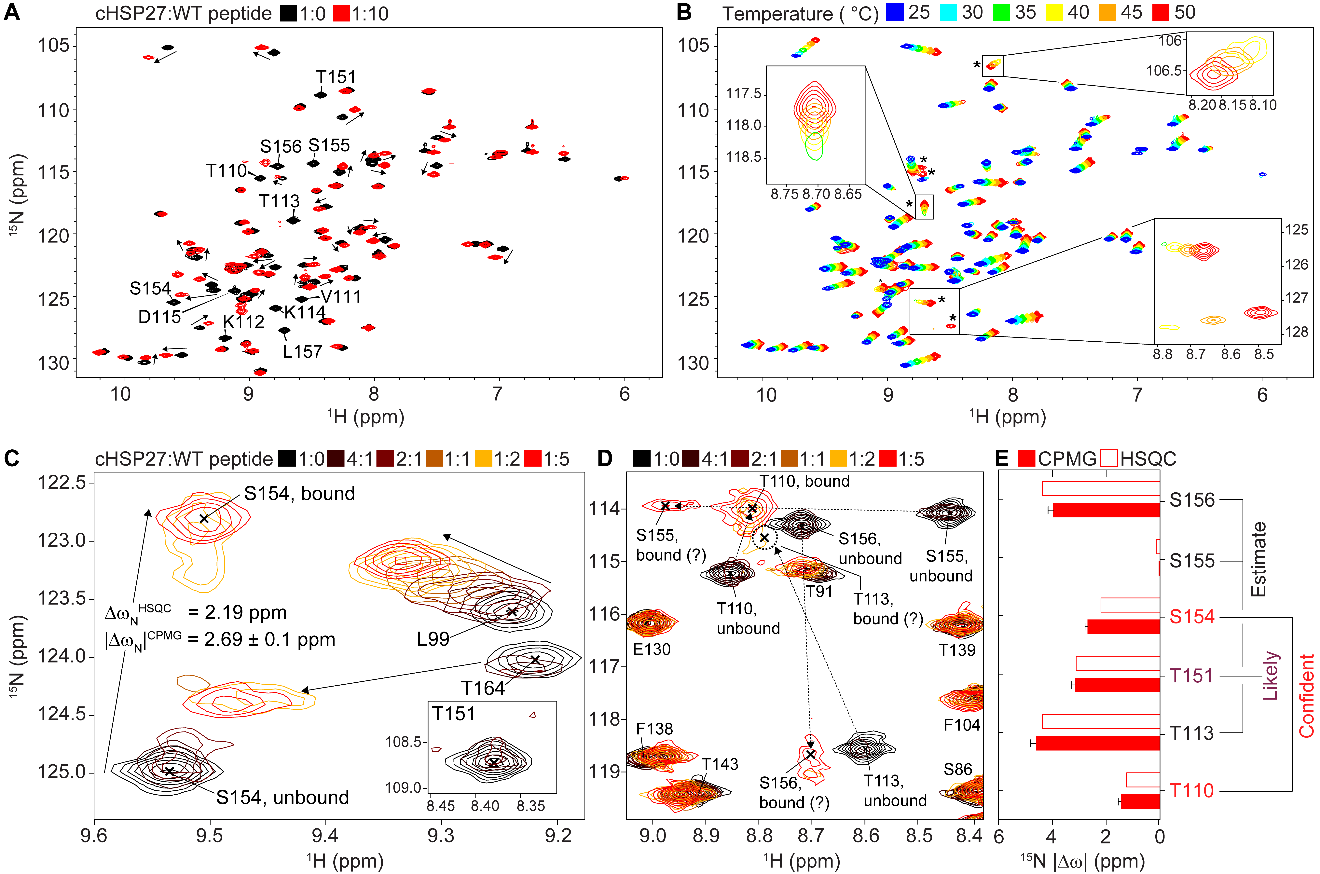


**Appendix Figure S6.** **Resonances in the β4/β8 groove disappear upon peptide binding and only reappear at higher temperatures.** (**A**) 2D ^1^H-^15^N HSQC spectra of ^15^N-cHSP27 in the absence (black) or presence (red) of a 10-fold molar excess of WT peptide (*ca.* 94% bound). Peaks that broaden are labeled. Arrows designate peaks that move but can be followed during the titration. Note that S154 can still be followed (*vide infra*). For broadened peaks that do not reappear in the presence of a 10-fold molar excess of peptide, the broadening is presumably caused by intermediate exchange between peptide-bound (major) and peptide-free (minor) forms. (**B**) For the sample with a 10-fold molar excess of WT peptide, increasing the temperature from 25 to 50 °C increases the exchange rate, thus shifting the exchange regime closer towards the fast exchange limit. The otherwise missing resonances can be recovered at higher temperatures (labeled with *). The insets show zoomed-in regions for a subset of these resonances. (**C**) Qualitative comparison of ^15^N chemical shift differences obtained by CPMG RD with those observed directly in HSQC spectra. Zoomed-in region of 2D ^1^H-^15^N spectra of ^15^N-cHSP27 at 25 °C in the absence (black) or presence of increasing amounts of WT peptide (brown-to-orange-to-red). While resonance assignments for the broadened resonances in the peptide-bound form of cHSP27 are not available, S154 can be followed throughout the titration. Peptide binding causes a ^15^N chemical shift change to S154 (2.2 ppm) that is consistent with that obtained by CPMG RD (2.7 ppm). Resonances from L99 and T164 are also labeled for clarity. Note that T164 is severely broadened during the titration due to a large ^1^H chemical shift difference between the peptide-free and -bound states. An inset shows the initial upfield movement of the T151 resonance. (**D**) The same spectra as panel (**C**) but focusing on a different region. The resonances from T110, T113, S155, and S156 in the β4/β8 groove broaden upon peptide binding. Note that resonance assignments for these residues in the peptide-bound state are not available. Based on the initial movement of resonances at early stages in the titration and the CPMG RD data, we estimated the resonance assignments of these residues. The initial (unbound) and final (bound) resonances are labeled; the “?” signifies that assignments are not available. The dashed circle at 8.8/114.7 ppm signifies the position of the assumed T113 bound resonance, based on the temperature titration above in panel (**B**). (**E**) Comparison of ^15^N chemical shift differences obtained by CPMG RD with those observed directly in the HSQC spectra of peptide-free and -bound cHSP27. Note that the assignments of peptide-bound cHSP27 are tentative and based on the initial movement of resonances during the early stages of the titration in combination with the CPMG RD data. The resonances from T110 and S154 could be followed throughout the titration and are therefore more confidently assigned (*Confident*). The resonance from T151 (*Likely*) is well-resolved and near resonances that can all be followed throughout the titration (D107, G132, G147, S154, G161, see panel **A**), and T151 moves slightly upfield in the ^15^N dimension before becoming broadened beyond detection (see panel **C**). This would suggest that the bound-state signal from T151 is upfield of its free signal in the ^15^N dimension. Therefore, T151 is tentatively assigned to the new signal that appears in the 50 °C spectrum at 8.17/106.6 ppm. The ^15^N chemical shift at 25 °C for this signal was derived from the observed temperature-dependence of the resonance (*ca.* -0.2 ppm in ^15^N per 5 °C, *i.e.* 105.6 ppm at 25 °C). For the other residues listed in the graph (*Estimate*), we estimated assignments and relied on our CPMG RD data. For T113, it moves slightly upfield in the ^15^N dimension at early stages in the titration. This suggests that the T113 bound state resonates upfield relative to its unbound signal. For S155, the CPMG RD data show that it yields a flat dispersion, indicative of a negligible ^15^N chemical shift difference upon peptide binding (labeled as ~0 ppm). Thus, the observed broadening must arise from a large ^1^H chemical shift change upon peptide binding. In addition, the early titration points show that S155 moves slightly downfield in the ^1^H dimension. The new resonance that appears at 8.98/114 ppm is consistent with the above assumptions. For S156, it moves slightly downfield at early titration points and exhibits a large ^15^N |Δω| value. The new peak near 8.7/119 ppm is consistent with the CPMG RD data. The remaining residues with large ^15^N |Δω| appear in a crowded region of the spectrum and are difficult to analyze without confident assignments.

**Appendix Figure S7**


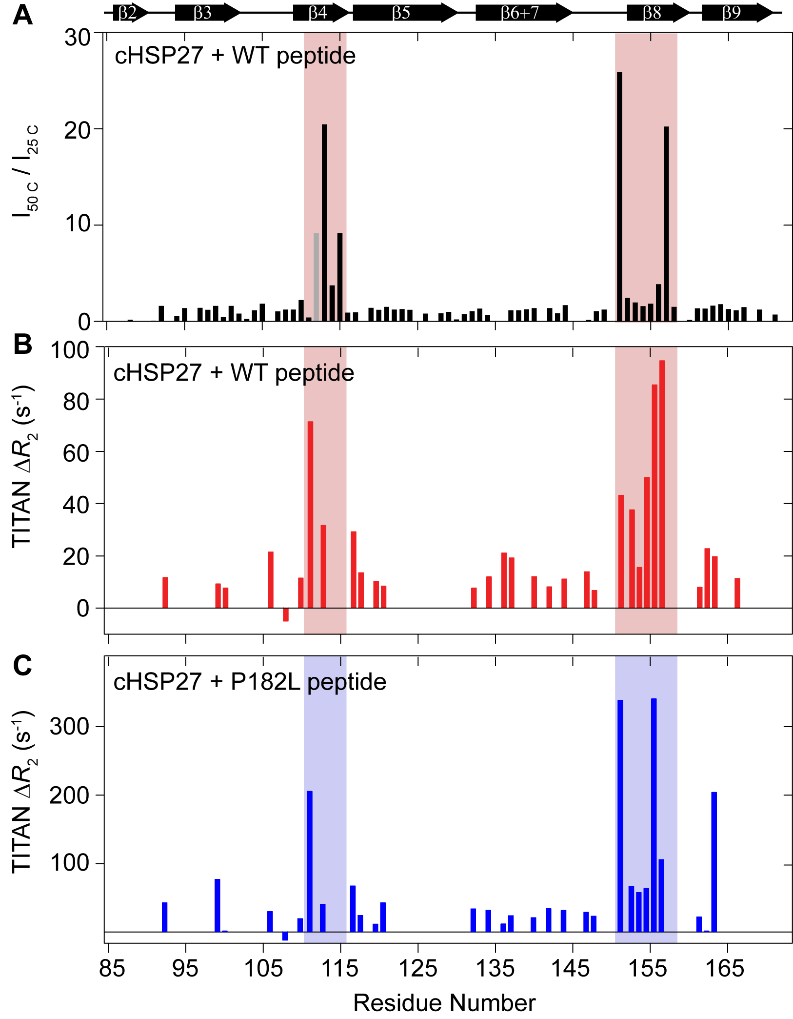


**Appendix Figure S7.** **Recovery of NMR signal intensities in peptide-bound cHSP27 at high temperature.** (**A**) 2D ^1^H-^15^N HSQC spectra were recorded on ^15^N-cHSP27 in the presence of a 10-fold molar excess of WT peptide at 25 °C and 50 °C at pH 7. The percentage of bound state is approximately 95% under these conditions based on a *K*_d_ of 125 µM. The ratio of peak intensities in the spectra are plotted with the intensity of the 50 °C spectrum divided by that in the 25 °C spectrum (I_50 C_ / I_25 C_). For residues in structured regions that are protected from solvent exchange, the anticipated increase in intensity at 50 °C approximately scales with the inverse of the change in correlation time (τ_c_), because the detected NMR signals scale with the transverse relaxation rate (*R*_2_) that is inversely related to τ_c_. This estimation yields an expected value of I_50 C_ / I_25 C_ ≈ τ_c_^25 C^ / τ_c_^50 C^ ≈ 1.8. Residues in solvent exposed regions will have lower values of I_50 C_ / I_25 C_ due to increased exchange with water, which deteriorates the signal at higher temperatures at this pH. Because the signals for residues in the β4/β8 groove remain broadened at 25 °C, this implies that the bound state is in conformational exchange with at least one other bound state. By raising the temperature, we recover these broadened signals (e.g., I_50 C_ / I_25 C_ >> 1.8) since the interconversion rate of the bound states is increased relative to the chemical shift differences (*k*_ex_ >> |Δω|), thus moving from intermediate to fast exchange. The gray bar for K112 indicates that its final chemical shift is not observed, perhaps due to overlap with another signal. Note that resonance assignments are not available for the peptide-bound state of cHSP27 (c.f. Appendix Figure S6), and these assignments are based on the available data. (**B**, **C**) The titration data (Figure 3, Appendix Figure S5) were fit to a two-state model of binding with the software TITAN. Shown here are the fitted Δ*R*_2_ rates (*R*_2,bound_ − *R*_2,free_) for cHSP27 in the presence of WT (B) and P182L (C) peptides. Both ^1^H and ^15^N *R*_2_ values were used in the calculation of Δ*R*_2_ (i.e., [bound ^1H^H *R*_2_ + bound ^15^N *R*_2_] – [free ^1H^H *R*_2_ + bound ^15^N *R*_2_]).

**Appendix Figure S8**

**
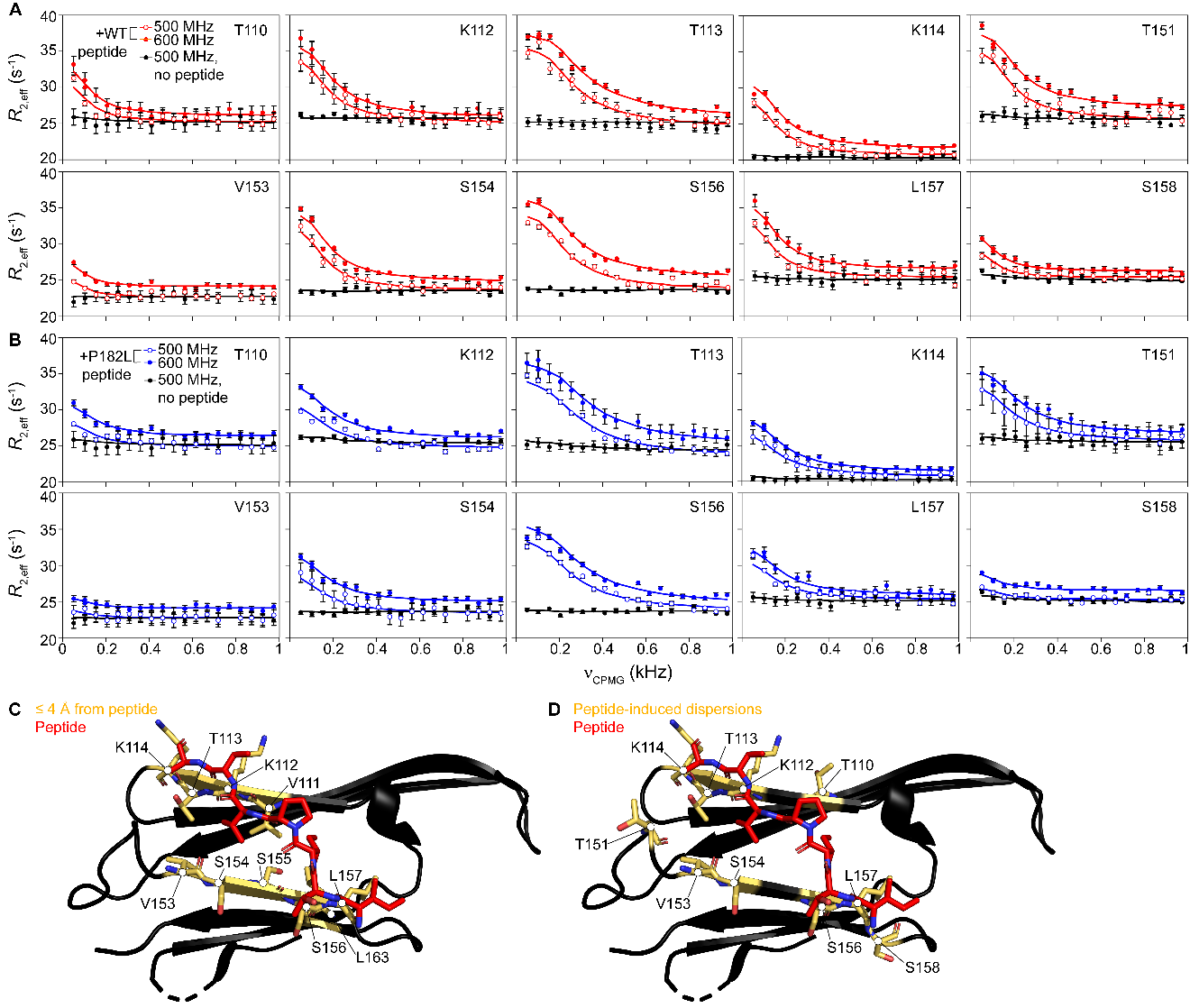
**

**Appendix Figure S8.** ^15^N CPMG RD data for ^2^H, ^15^N-cHSP27 in the presence of (**A**) WT and (**B**) P182L peptides. Dispersions at both 600 (solid) and 500 MHz (empty circles) are shown, with the solid lines indicating fits to the data. Errors are generated from *R*_2,eff_ values recorded at duplicate ν_CPMG_ fields. The black data points correspond to cHSP27 in the absence of peptide and are shown at 500 MHz only. Residue numbers are indicated in the upper-right of each panel. (**C**) The crystal structure of peptide-bound cHSP27 (PDB: 4mjh) rotated to show the β4/β8 groove. Only a single subunit of the cHSP27 dimer is shown here. The peptide is colored red and residues that have any atoms ≤ 4 Å from any atom in the peptide are colored yellow. (**D**) The same view as panel C but residues with dispersions in panels A and B are colored yellow. Note that the crystal structure (PDB: 4mjh) does not have any electron density for residue E186, and only some electron density for the side chain of F185, which may explain why the nearby T151 residue yields a relatively large Δω despite not meeting the 4-Å threshold. Likewise, the residue S158, which also does not meet the 4-Å threshold, likely senses the N-terminal residue (E178) of the peptide, which was not present in the peptide used for crystallization.

**Appendix Figure S9**


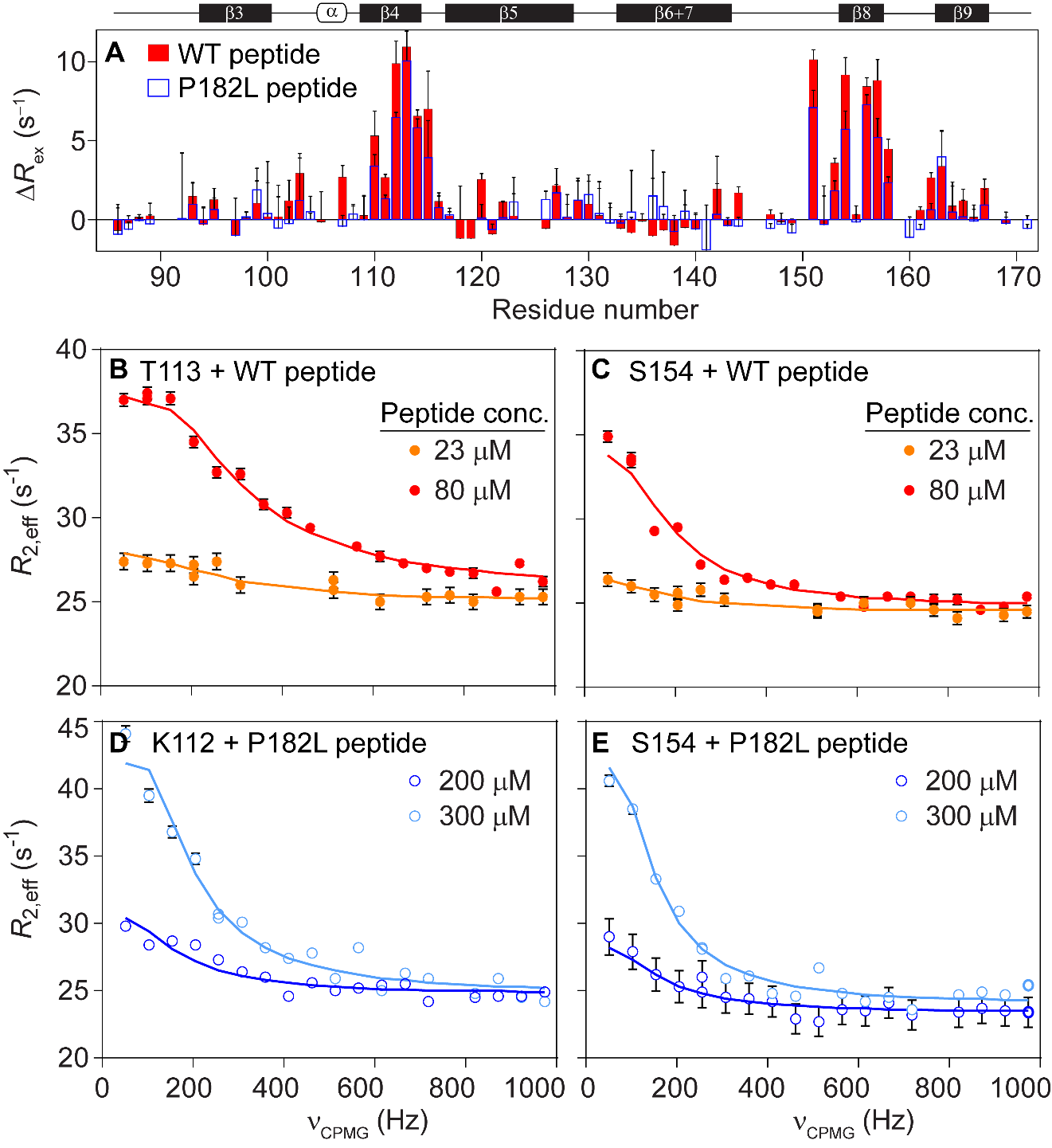


**Appendix Figure S9. Concentration-dependence of dispersions from peptide-bound cHSP27.** (**A**) *R*_ex_ is defined as the difference in *R*_2,eff_ values recorded at low and high ν_CPMG_ fields. Shown here is the difference in *R*_ex_ (Δ*R*_ex_ = *R*_ex_^presence^ - *R*_ex_^absence^) between cHSP27 in the presence and absence of WT (red) or P182L (blue) peptides. The samples contained *ca.* 2% of the peptide-bound forms. Positive values of Δ*R*_ex_ suggest increased millisecond motions due to exchange between the peptide-free and peptide-bound forms. (**B**, **C**) ^15^N CPMG RD data for a residue in the β4 strand (T113) and the β8 strand (S154) in the presence of *ca.* 2% (red) or *ca*. 0.2% (orange) cHSP27-WT peptide complex. (**D, E**) Dispersions from residues in the β4 or β8 strand as a function of added P182L peptide. In dark blue is *ca.* 2% of the cHSP27-P182L peptide complex and in light blue is *ca*. 4% of the cHSP27-P182L peptide complex.

**Appendix Figure S10**


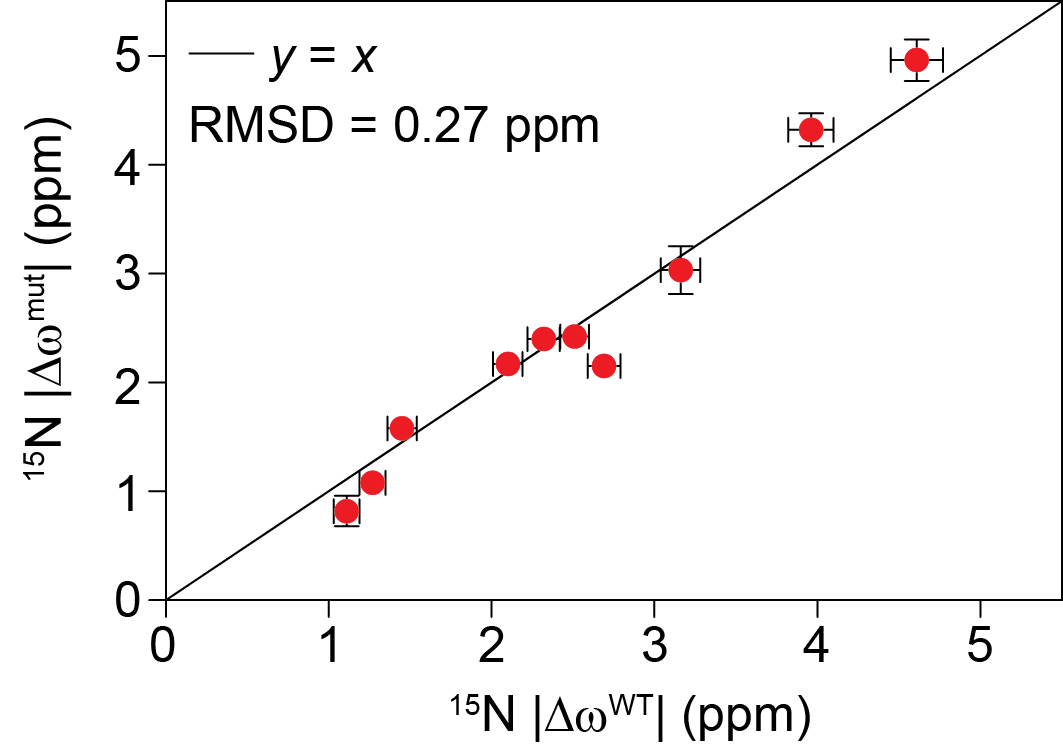


**Appendix Figure S10.** **Similarity of the WT and mutant peptide-bound states.** The overall similarity between the ^15^N |Δω| values for the ACD in the presence of WT or P182L peptides (RMSD = 0.27 ppm), suggests that cHSP27 adopts a relatively similar conformation when bound to the WT or P182L peptide. The superscript ^mut^ refers to P182L. Note that most of these resonances were severely broadened in the peptide titrations in Fig. 3 of the main text, and thus their CSPs could not be directly measured. CPMG RD, however, enabled measurement of their ^15^N |Δω| values upon peptide binding.

**Appendix Figure S11**


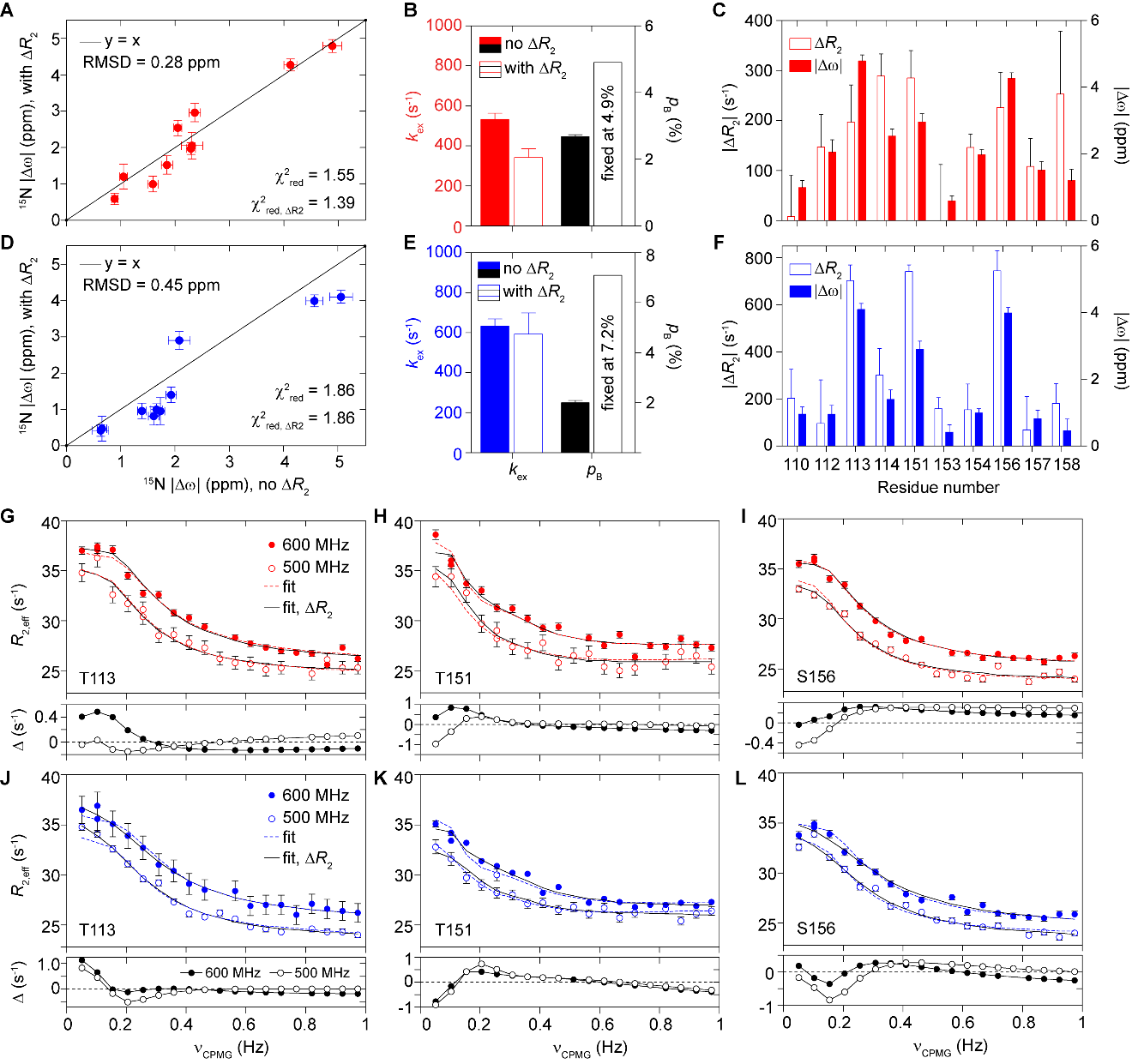


**Appendix Figure S11.** **Global analysis of the dispersion and titration data.** (**A**) Correlation plot showing the fitted ^15^N |Δω| values in CPMG RD experiments on ^2^H/^15^N-cHSP27 in the presence of WT peptide. The *x* axis shows the ^15^N |Δω| values obtained from a standard fit to a two-state model with Δ*R*_2_ (Δ*R*_2_ = *R*_2,A_ – *R*_2,B_) set to zero, and the *y* axis shows the fit performed with *p*_B_ fixed at the value expected based on a *K*_d_ of 125 µM (4.9%) and Δ*R*_2_ (Δ*R*_2_ = *R*_2,A_ – *R*_2,B_) allowed to be nonzero. The RMSD is shown in the upper-left corner. The reduced χ^2^ values for the two fitting approaches are shown in the lower-right corner with χ^2^_red_ corresponding to the naïve two-state model and χ^2^_red, ΔR2_ corresponding to the fit with *p*_B_ fixed at 4.9% and Δ*R*_2_ ≠ 0. (**B**) The fitted *k*_ex_ and *p*_B_ values from panel A for the two fitting approaches. Note that *p*_B_ was fixed at 4.9% in the Δ*R*_2_ ≠ 0 fitting approach. (**C**) The fitted values of Δ*R*_2_ shown alongside the fitted ^15^N |Δω| values. (**D**-**F**) the same as panels A-C except for the CPMG RD experiments on ^2^H/^15^N-cHSP27 in the presence of the P182L peptide with *p*_B_ fixed at 7.2%. (**G**-**L**) *Top*: ^15^N dispersions from ^2^H/^15^N-cHSP27 in the presence of the WT or P182L peptide for residues T113 (**G**, **J**), T151 (**H**, **K**), and S156 (**I**, **L**). Data at 600 and 500 MHz are respectively shown with filled and empty circles. The best-fit values for the standard, 2-state model are shown in dashed lines, whereas the global model with fixed *p*_B_ and a non-zero Δ*R*_2_ value are shown in solid lines. *Bottom*: the difference between the best-fit values at 600 (filled) and 500 MHz (empty circles), with Δ referring to the values from the global model minus the values from the standard 2-state model.

**Appendix Figure S12**


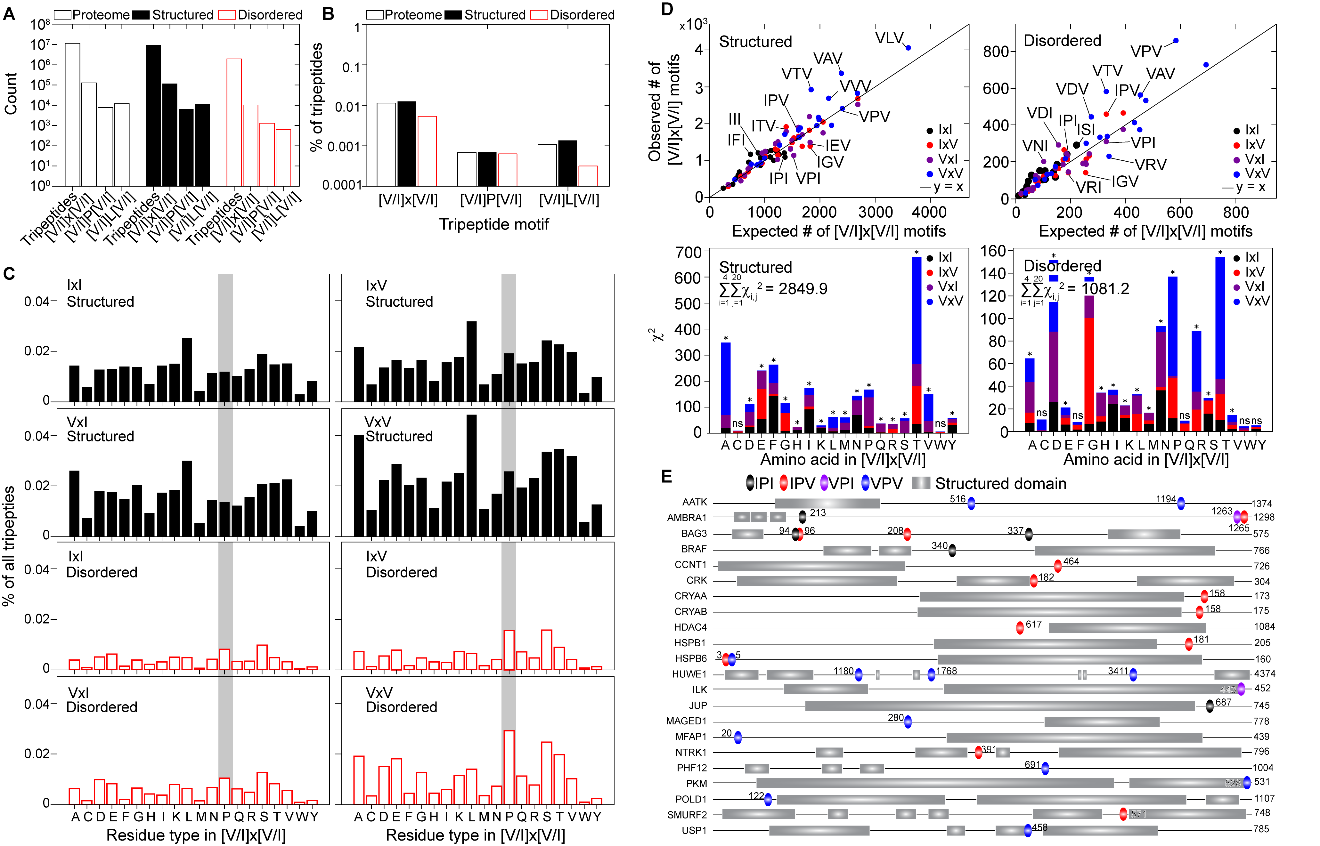


**Appendix Figure S12.** **Bioinformatics analyses of [V/I]x[V/I] motifs in the proteome.** (**A**) The total number of tripeptides and [V/I]x[V/I], [V/I]P[V/I], and [V/I]L[V/I] motifs in (empty black bars) the proteome, (black bars) structured regions of the proteome, and (empty red bars) disordered regions of the proteome. (**B**) The percentage of [V/I]x[V/I], [V/I]P[V/I], and [V/I]L[V/I] motifs for the proteome, structured regions, and disordered regions normalized to the total number of tripeptides present in each. (**C**) The distribution of [V/I]x[V/I] motifs as a function of the central residue X for all of the possible combinations of [V/I]x[V/I]. Structured regions are shown in black bars and disordered regions in empty red bars. Proline is indicated with a grey box to denote the [V/I]P[V/I] motif. (**D**) The observed number of IxI, IxV, VxI, and VxV motifs in structured regions of the proteome compared against the expected number of such motifs calculated using the frequency of amino acids in structured regions. The same plot is included for disordered regions using the frequencies of amino acids in disordered regions. For the structured and disordered plots shown here, the *R*^2^ values from a linear regression (not plotted) are respectively 0.89 and 0.86 with slopes of 0.83 and 1.12. Bottom panel: stacked bar graphs showing reduced χ^2^ values for each residue type for each motif (IxI, IxV, VxI, VxV) for structured (left) or disordered (right) regions. The total χ^2^ value, summed over each residue type (*j* = 1, 2, 3, … , 20) and each motif (*i* = 1, 2, 3, 4) is shown in the upper left region. The χ^2^ values are highly significant (*p* < 0.001) for this system with ν = (20-1) x 4 = 76 total degrees of freedom, which indicates that the null hypothesis – that the frequency of [V/I]x[V/I] motifs is described solely by amino acid frequencies – is not correct. Rather, other factors, most likely some forms of evolutionary selection, have led to a non-random distribution of [V/I]x[V/I] motifs. For each residue type, statistical significance was inferred from the summed χ^2^ value across the four motifs (ν = 3). Residue types with an asterisk (*) indicate a *p* value < 0.05, with most having *p* values < 0.001. The “ns” symbol denotes not significant, *i.e.* *p* value > 0.05. (**E**) Linear domain depiction of the 22 proteins that both interact with HSP27 and contain [V/I]x[V/I] motifs. The location of their [V/I]P[V/I] SLiMs and structured domains are indicated, along with their length. The numbers next to the [V/I]P[V/I] SLiMs indicate the starting residue number of the motif.

**Appendix Figure S13**


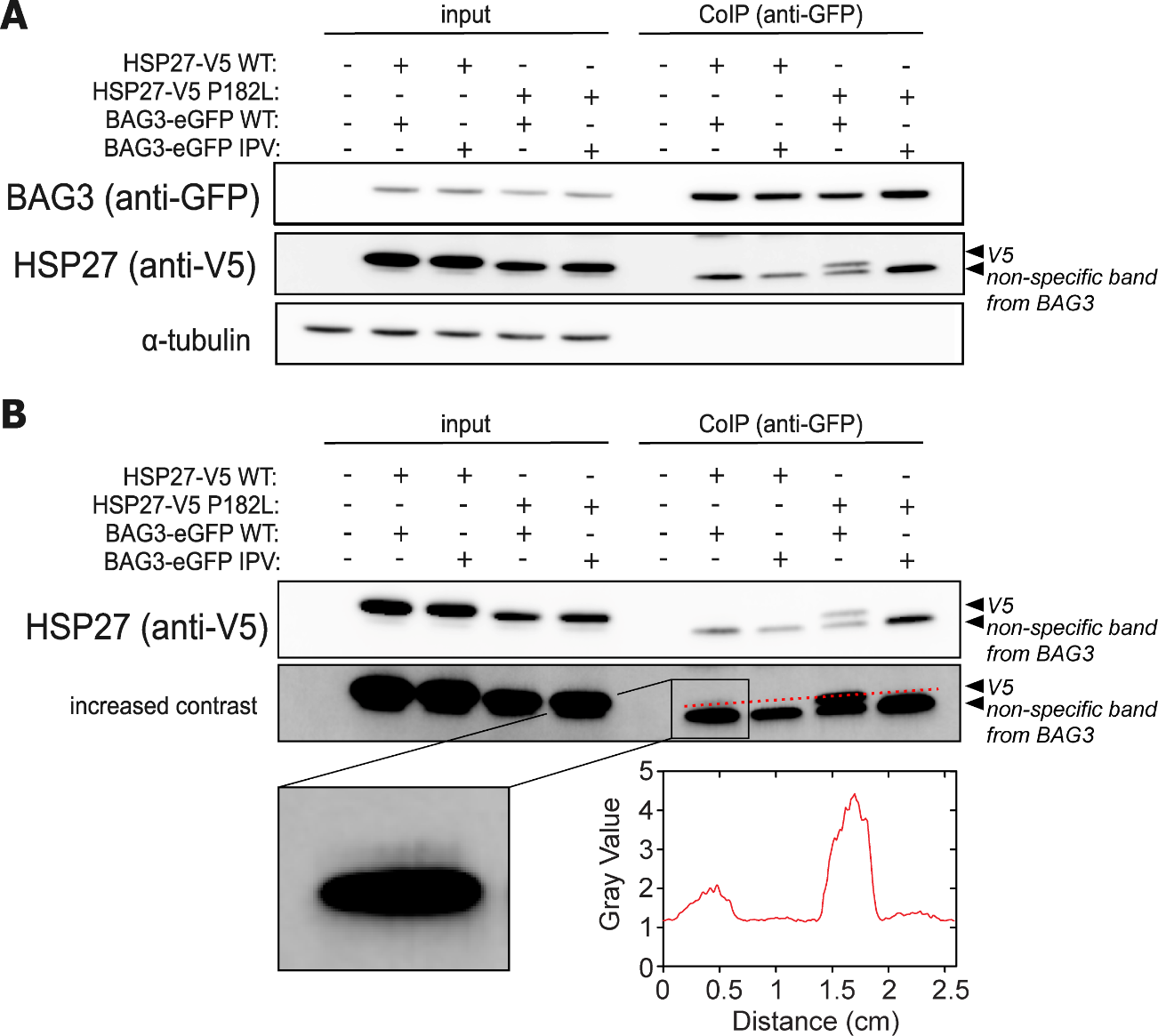


**Appendix Figure S13. Co-immunoprecipitation of BAG3 by WT HSP27.** (**A**) The same western blot as Figure 5E in the main text. See legend of Figure 5E for details. While WT HSP27 pulls down BAG3, a clear band from BAG3 is not immediately apparent in this blot. (**B**) The same western blot as panel A, with the anti-V5 row shown at increased contrast where the band from BAG3 becomes evident in the HSP27-V5 WT and BAG3-eGFP WT lane, which can also be observed on the line intensity profile (lower right). The line used to obtain the intensity profile is drawn on the blot (dashed red line) along the V5 band positions. The lower left depicts a zoomed-in region of the WT HSP27 lane, where the band from BAG3 is visible. Note that panels A and B are the same western blot.

**Appendix Figure S14**


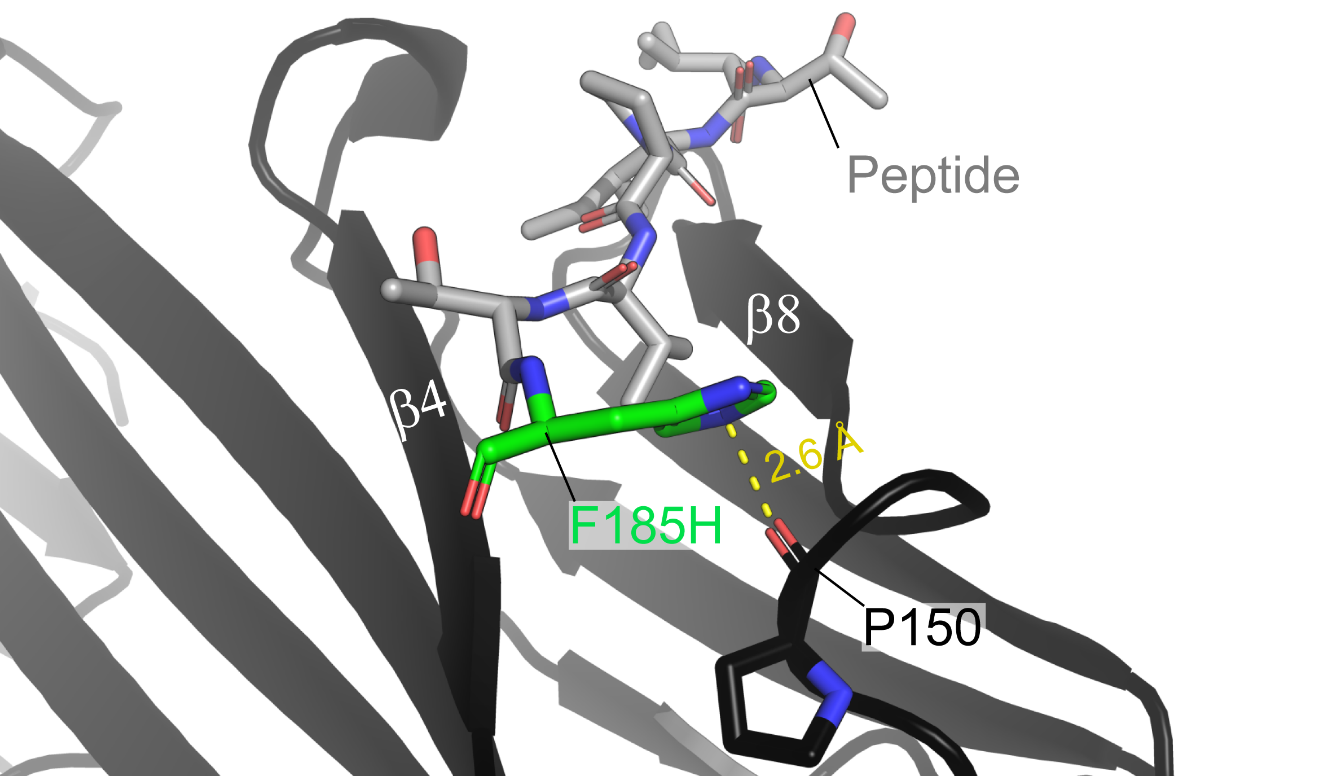


**Appendix Figure S14**. **Interactions between the non-IxI/V residues in the peptide and the ACD**. The F185H mutation significantly increased the binding affinity of the IxI/V peptide for the ACD ([Freilich *et al*, 2018](#References)). This mutation could create a new hydrogen bond between the H185 side chain and the P150 carbonyl. The mutation was introduced into the crystal structure of cHSP27 bound to the IxI/V-bearing peptide (PDB: 4mjh) using the PyMol mutagenesis tool. The peptide is colored grey, the F185H mutation is colored green, and the ACD is colored black. The β4 and β8 strands are labeled. Residues that are shown in sticks are colored by element type (oxygen is red and nitrogen is blue). The 2.6-Å distance was calculated from the coordinates of the H185(N^ε2^) and P150(CO) atoms, and it does not take into account the otherwise absent H^ε2^ proton or the distance of the N^ε2-^H^ε2^ bond. The H185 rotamer was selected manually and does not reflect an energy-minimized conformation.

**Tables**

Appendix Table S1. ^15^N |Δω| values for WT- and P182L-bound cHSP27. ^15^N |Δω| values in cHSP27 derived from CPMG RD analysis in the presence of a small amount of WT or P182L peptide. Dispersions at 25 °C at 500 and 600 MHz were included in the global fit; WT and P182L datasets were analyzed independently. The superscripts ^WT^ and ^P182L^ refer to values obtained in the presence of WT peptide and P182L peptide, respectively. Errors reflect the uncertainty as obtained from the covariance matrix of the fit.

| **Residue** | **^15^N** \|Δω\| **^WT^ (ppm)** | **Error^WT^ (ppm)** | **^15^N** \|Δω\|**^P182L^ (ppm)** | **Error^P182L^ (ppm)** |
| --- | --- | --- | --- | --- |
| T110 | 1.45 | 0.09 | 1.58 | 0.07 |
| K112 | 2.32 | 0.10 | 2.40 | 0.08 |
| T113 | 4.61 | 0.16 | 4.96 | 0.19 |
| K114 | 2.51 | 0.09 | 2.42 | 0.09 |
| T151 | 3.16 | 0.12 | 3.03 | 0.22 |
| V153 | 1.11 | 0.08 | 0.82 | 0.14 |
| S154 | 2.69 | 0.10 | 2.15 | 0.10 |
| S156 | 3.96 | 0.14 | 4.32 | 0.15 |
| L157 | 2.10 | 0.09 | 2.17 | 0.09 |
| S158 | 1.27 | 0.08 | 1.08 | 0.07 |

Appendix Table S2. Kinetic parameters for WT and P182L peptide binding to cHSP27. Values of *k*_on_ (M^-1^ s^-1^), *k*_off_ (s^-1^), and *K*_d_ (µM) as derived from the titration and CPMG RD analyses on peptide-bound cHSP27. The *K*_d_ refers to the dissociation constant of peptide binding to cHSP27, with *k*_off_ and *k*_on_ the dissociation and association rates, respectively. CSP, CPMG, CPMG Δ*R*_2_, and TITAN respectively refer to values determined using CSPs from fast-exchange resonances, CPMG RD with a two-state fit, CPMG RD using a fixed *p*_B_ at the value expected from the *K*_d_ and Δ*R*_2_ ≠ 0, or lineshape analysis with TITAN. Errors from CSP, CPMG, and CPMG Δ*R*_2_ reflect the uncertainty as obtained from the covariance matrix of the fit. Errors from TITAN were obtained through a χ^2^ analysis in which either *k*_off_ or *K*_d_ was fixed while the other was fitted. The value of the fixed parameter was iterated, with the value of the resultant χ^2^ tabulated to compute the probability distribution: exp(-(χ^2^ - χ^2^_min_)/2). Because TITAN fits *k*_off_ and *K*_d_, with *k*_on_ computed from the known relation (*K*_d_ = *k*_off_/*k*_on_), we obtained the error surface for *k*_on_ by fixing *k*_off_ at the best-fit value while *K*_d_ was iterated. The values listed within square brackets correspond to a parameter with a non-Gaussian χ^2^ distribution. The listed values are the minimum and maximum values that have values of exp(-(χ^2^ - χ^2^_min_)/2) ≥ 0.6. The *k*_ex_ value for TITAN was calculated assuming the same conditions used for CPMG RD (1.5 mM protein, 80 or 200 μM peptide) and the equation *k*_ex_ = *k*_off_ + *k*_on_[L]_free_, with [L]_free_ derived from the *K*_d_ and known relations (see Methods).

| **Pep.** | **Dataset** | ***K*_d_ (µM)** | ***k*_off_ (s^-1^)** | ***k*_on_ (M^-1^ s^-1^)** | ***p*_B_ (%)** | ***k*_ex_ (s^-1^)** |
| --- | --- | --- | --- | --- | --- | --- |
| WT | CSP  TITAN  CPMG  CPMG Δ*R*_2_ | 124 ± 13  132 ± 58  1,762 ± 120  124 (fixed) | ----  207 ± 85  515 ± 33  346 ± 25 | ----  1.6 ± 0.7 x 10^6^  2.9 ± 0.1 x 10^5^  2.8 ± 0.2 x 10^6^ | 4.9 ± 0.5  4.9 ± 2.2  2.4 ± 0.1  4.9 (fixed) | ----  217 ± 130  528 ± 30  364 ± 21 |
| P182L | CSP  TITAN  CPMG  CPMG Δ*R*_2_ | 1030 ± 118  1285 [700, 2000]  8,373 ± 534  1030 (fixed) | ----  771 [350, 1750]  610 ± 39  547 ± 41 | ----  6.0 [3.8, 9.1] x 10^5^  7.3 ± 0.2 x 10^4^  5.0 ± 0.2 x 10^5^ | 7.2 ± 0.7  7.0 [5.5, 8.8]  2.0 ± 0.1  7.2 (fixed) | ----  828 [386, 1836]  622 ± 35  590 ± 33 |

Appendix Table S3. Energetic parameters for WT and P182L peptide binding to cHSP27. The differences in free energy (ΔΔG = ΔG_WT_ – ΔG_P182L_) are shown for the fitted values of *K*_d_, *k*_off_, and *k*_on_. All units are in kcal mol^-1^ with the calculations performed using a temperature of 298.15 K. The datasets CSP, TITAN, CPMG, and CPMG Δ*R*_2_ respectively refer to values determined from fitting fast-exchange resonances, fast- and slow-exchange resonances via TITAN, CPMG RD with a two-state fit, and CPMG RD using a fixed *p*_B_ at the value expected from the *K*_d_ and Δ*R*_2_ ≠ 0. The values listed within square brackets correspond to a parameter with a non-Gaussian χ^2^ distribution and are the minimum and maximum values that have values of exp(-(χ^2^ - χ^2^_min_)/2) ≥ 0.6.

| **Dataset** | **ΔΔ*G K*_d_** | **ΔΔ*G k*_off_** | **ΔΔ*G k*_on_** |
| --- | --- | --- | --- |
| CSP  TITAN  CPMG  CPMG Δ*R*_2_ | −1.25 ± 0.19  −1.34 [0.49, -2.29]  −0.92 ± 0.09  −1.25 | ----  −0.78 [-0.24, -1.84]  −0.10 ± 0.01  −0.27 ± 0.03 | ----  0.58 [0.37, 0.88]  0.82 ± 0.06  1.02 ± 0.08 |

Appendix Table S4. Proteome-wide analysis of IxI/V motifs. The percentages shown in the final column were calculated with respect to ^a^ the proteome, ^b^ the total number of tripeptides in the listed databases, or ^c^ the total number of [V/I]x[V/I] tripeptides. Disordered refers to both intrinsically disordered proteins and intrinsically disordered regions longer than 20 residues.

| **Motif** | **Database** | **Count** | **%** |
| --- | --- | --- | --- |
| XXX ^a^ | Proteome | 11,373,813 | – |
|  | Structured | 8,427,904 | – |
|  | Disordered | 2,945,909 | – |
| [V/I]x[V/I] ^b^ | Proteome | 128,604 | 1.1% |
|  | Structured | 112,961 | 1.3% |
|  | Disordered | 15,643 | 0.5% |
| [V/I]P[V/I] ^c^ | Proteome | 7,803 | 6.1% |
|  | Structured | 5,935 | 5.3% |
|  | Disordered | 1,868 | 11.9% |
| [V/I]L[V/I] ^c^ | Proteome | 12,309 | 9.6% |
|  | Structured | 11,372 | 10.1% |
|  | Disordered | 937 | 6.0% |

Appendix Table S5. Known HSP27-interacting proteins that contain [I/V]-P-[I/V] motifs*.* For each hit, the gene name, UniProt ID, protein name, [I/V]-P-[I/V] composition and numbering, and the number of unique interactors in the BioGrid are listed. Only [I/V]-P-[I/V] motifs in disordered regions were considered.

| **Gene** | **UniProt ID** | **Protein** | **[I/V]-P-[I/V]** | **Interactors** |
| --- | --- | --- | --- | --- |
| *AATK* | Q6ZMQ8 | Apoptosis-associated tyrosine kinase | ^516^VPV^518^  ^1194^VPV^1196^ | 53 |
| *AMBRA1* | Q9C0C7 | Activating molecule in Beclin-1-regulated autophagy | ^213^IPI^215^  ^1263^VPI^1265^  ^1265^IPV^1267^ | 212 |
| *BAG3* | O95817 | Bcl-2-associated anthanogene-3 | ^94^IPI^96^  ^96^IPV^98^  ^208^IPV^210^  ^337^IPI^339^ | 504 |
| *BRAF* | P15056 | serine/threonine-protein kinase B-Raf | ^340^IPI^342^ | 123 |
| *CCNT1* | O60563 | Cyclin-T1 | ^464^IPV^466^ | 109 |
| *CRK* | P46108 | proto-oncogene c-Crk | ^182^IPV^184^ | 265 |
| *CRYAA* | P02489 | αA-crystallin (HSPB4) | ^158^IPV^160^ | 68 |
| *CRYAB* | \| P02511 \| \| --- \| | αB-crystallin (HSPB5) | ^158^IPI^160^ | 119 |
| *HDAC4* | P56524 | Histone deacetylase 4 | ^617^IPV^619^ | 353 |
| *HSPB1* | P04792 | HSP27 (HSPB1) | ^181^IPV^183^ | 452 |
| *HSPB6* | O14558 | HSP20 (HSPB6) | ^3^IPV^5^  ^5^VPV^7^ | 10 |
| *HUWE1* | Q7Z6Z7 | E3 ubiquitin-protein ligase HUWE1 | ^1180^VPV^1182^  ^1768^VPV^1170^  ^3411^VPV^3413^ | 519 |
| *ILK* | Q13418 | Integrin-linked kinase | ^443^VPI^445^ | 238 |
| *JUP* | P14923 | Junction plakoglobin (gamma-catenin) | ^687^IPI^689^ | 223 |
| *MAGED1* | Q9Y5V3 | Melanoma-associated antigen D1 | ^280^VPV^282^  ^490^VPI^492^ | 143 |
| *MFAP1* | P55081 | Microfibrillar-associated protein 1 | ^20^VPV^22^ | 115 |
| *NTRK1* | P04629 | Neurotrophic receptor tyrosine kinase | ^391^IPV^393^ | 1960 |
| *PHF12* | Q96QT6 | PHD finger 12 | ^619^VPV^621^ | 39 |
| *PKM* | P14618 | Pyruvate kinase M | ^528^VPV^530^ | 235 |
| *POLD1* | P28340 | DNA polymerase delta catalytic subunit | ^122^VPV^124^ | 125 |
| *SMURF2* | Q9HAU4 | E3 ubiquitin-protein ligase SMURF2 | ^571^IPV^573^ | 149 |
| *USP1* | O94782 | Ubiquitin carboxy-terminal hydrolase 1 | ^458^VPV^460^ | 74 |

**Table of Contents Graphic**


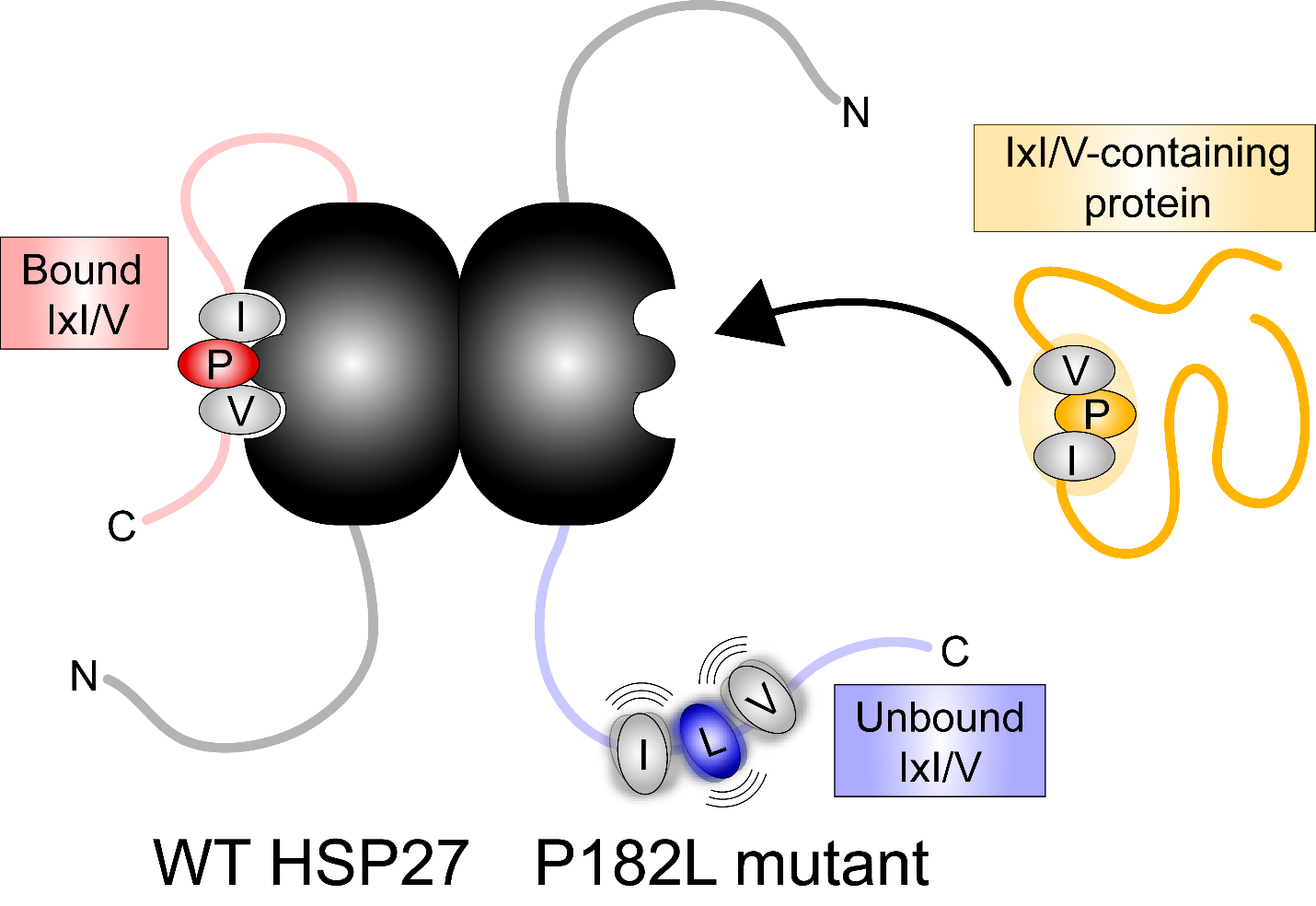

Supplement: Supplementary file 2 — Appendix [file EMBJ-40-e103811-s002.docx]
