# Supplementary figures and images for: A weakened interface in the P182L variant of HSP27 associated with severe Charcot‐Marie‐Tooth neuropathy causes aberrant binding to interacting proteins
Source: EMBO J. 2021 Mar 1;40(8):e103811. doi: 10.15252/embj.2019103811 (PMC8047445; doi:10.15252/embj.2019103811)

## Appendix Figure S3D

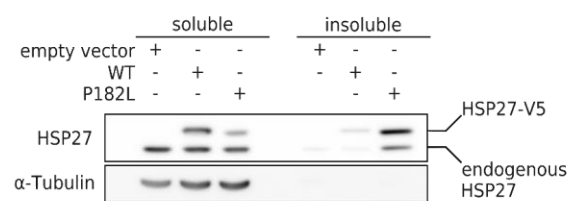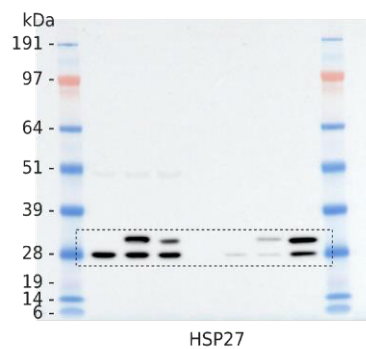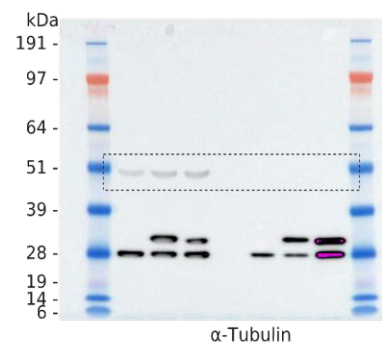

Supplement: Supplementary file 3 — Source Data for Appendix [file EMBJ-40-e103811-s001.zip › Appendix_Figure_Source_Data/Source_Data_Appendix_Figure_S3.pdf]

Appendix Figure S4B

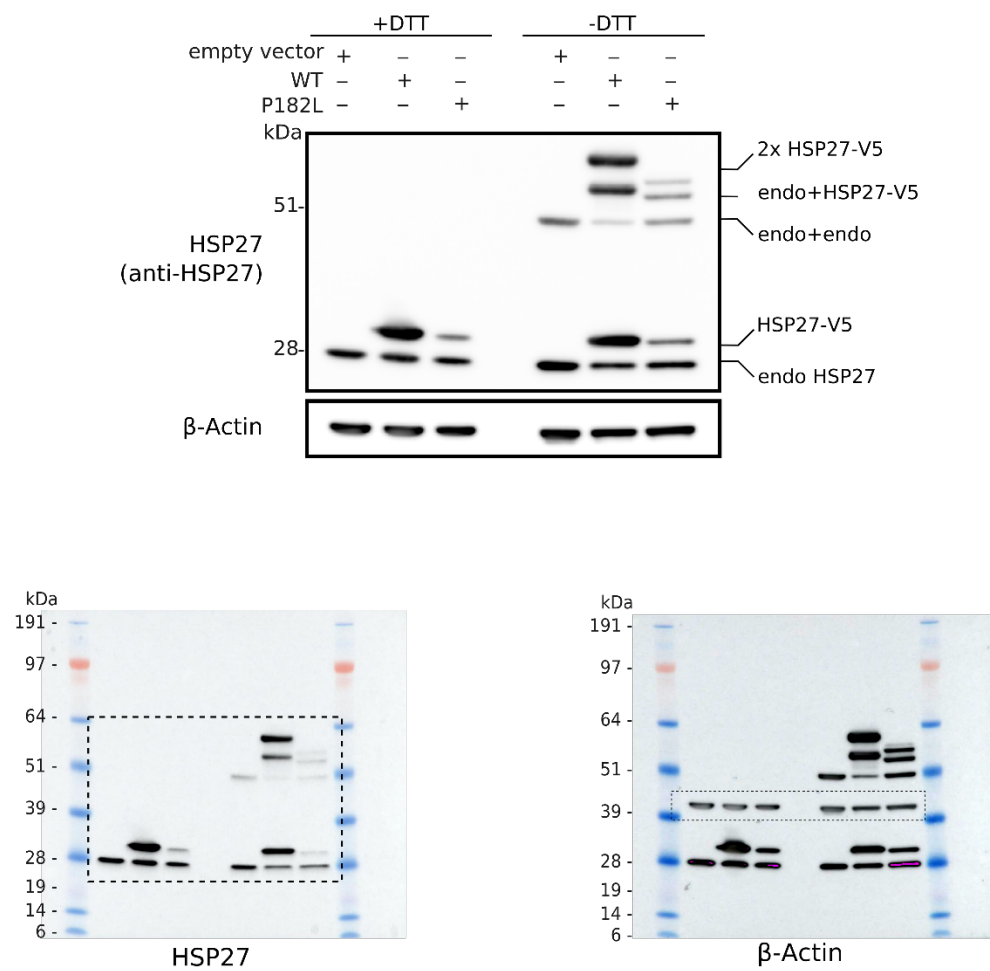

Appendix Figure S4C

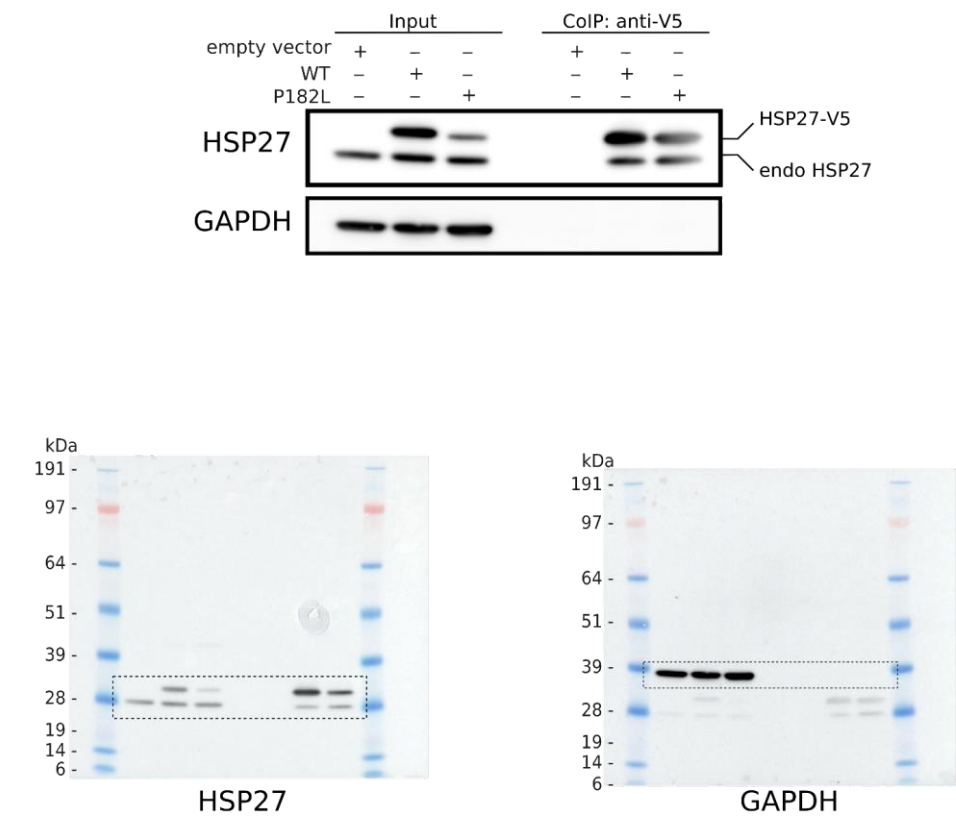

Supplement: Supplementary file 3 — Source Data for Appendix [file EMBJ-40-e103811-s001.zip › Appendix_Figure_Source_Data/Source_Data_Appendix_Figure_S4.pdf]

**Figure 2B**

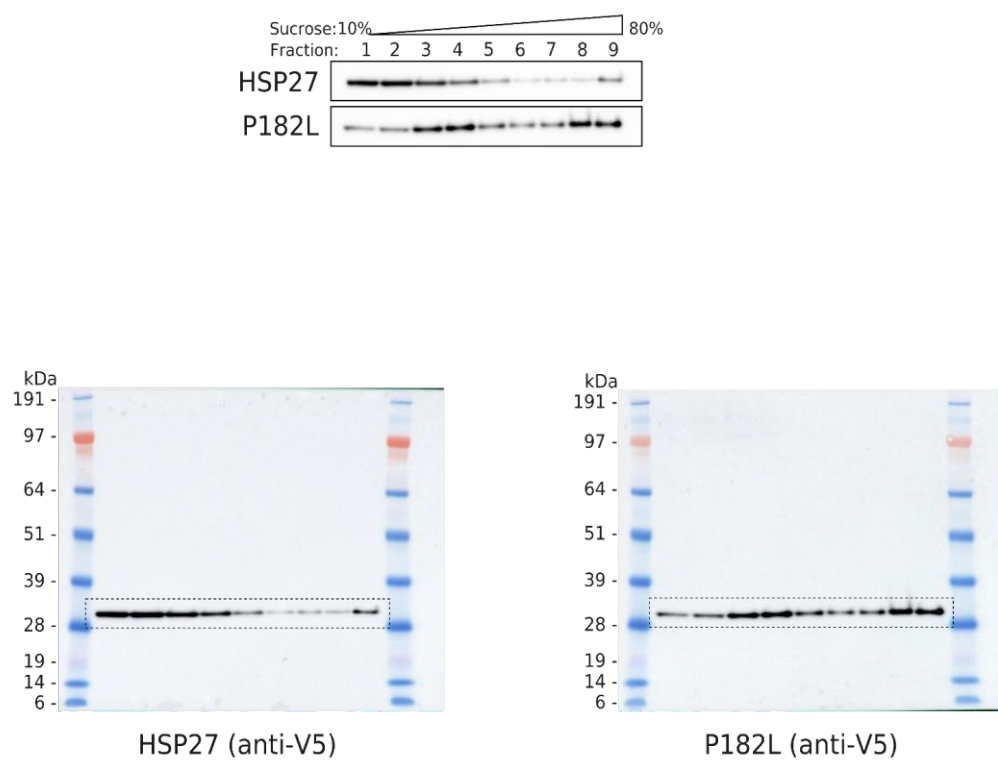

Supplement: Supplementary file 4 — Source Data for Figure 2 [file EMBJ-40-e103811-s004.pdf]

Figure 5D

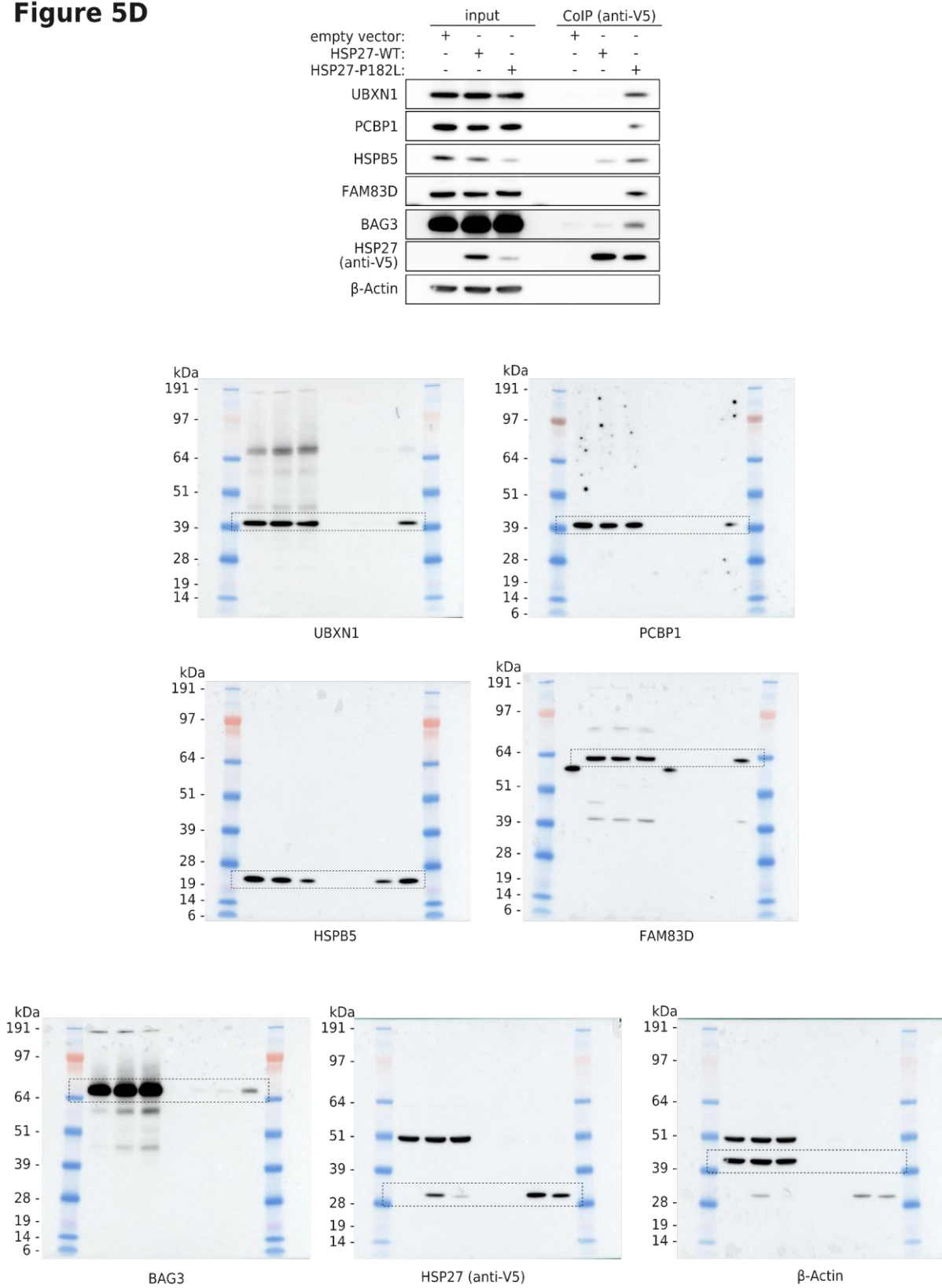

Figure 5E

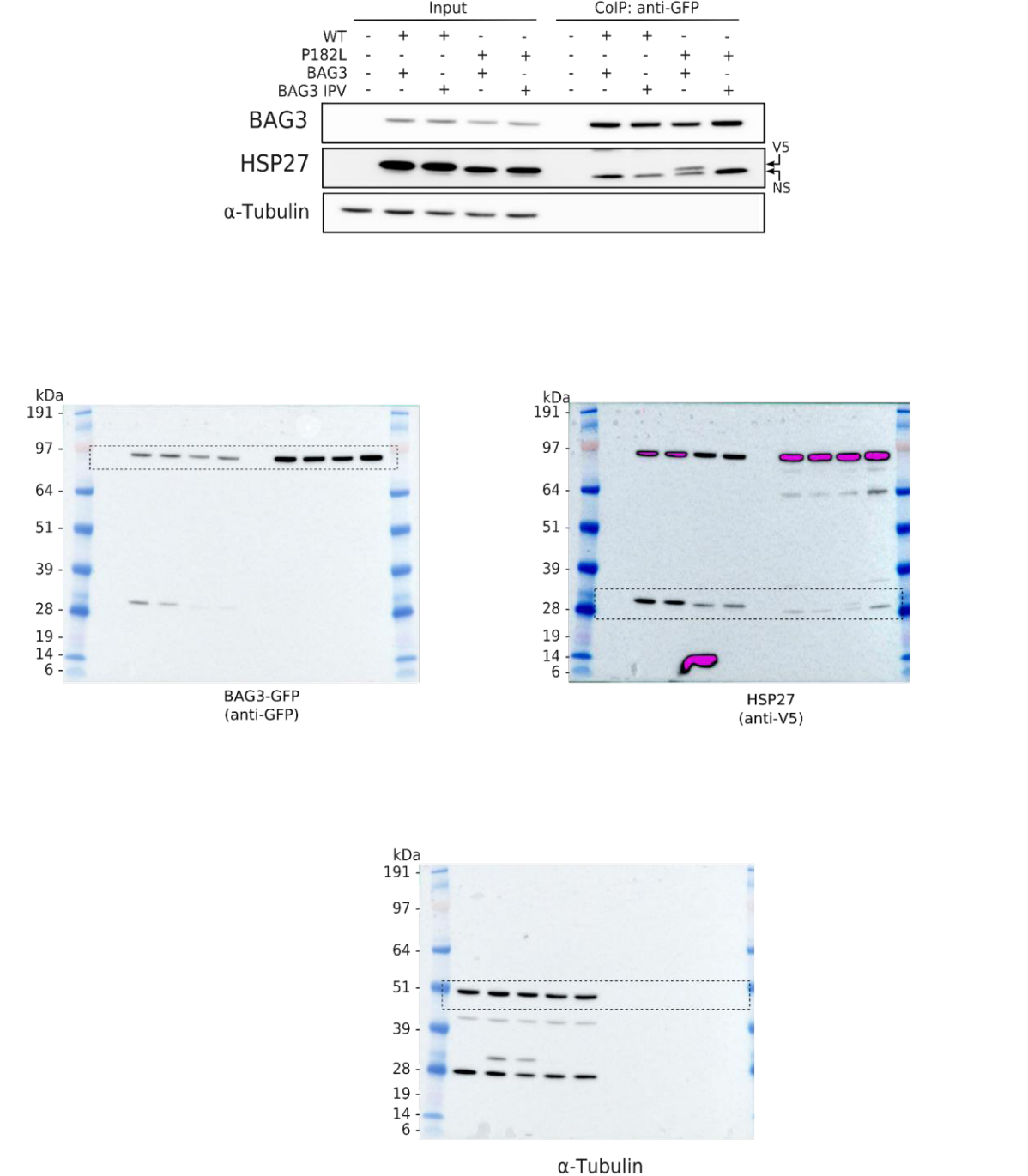

Figure 5F

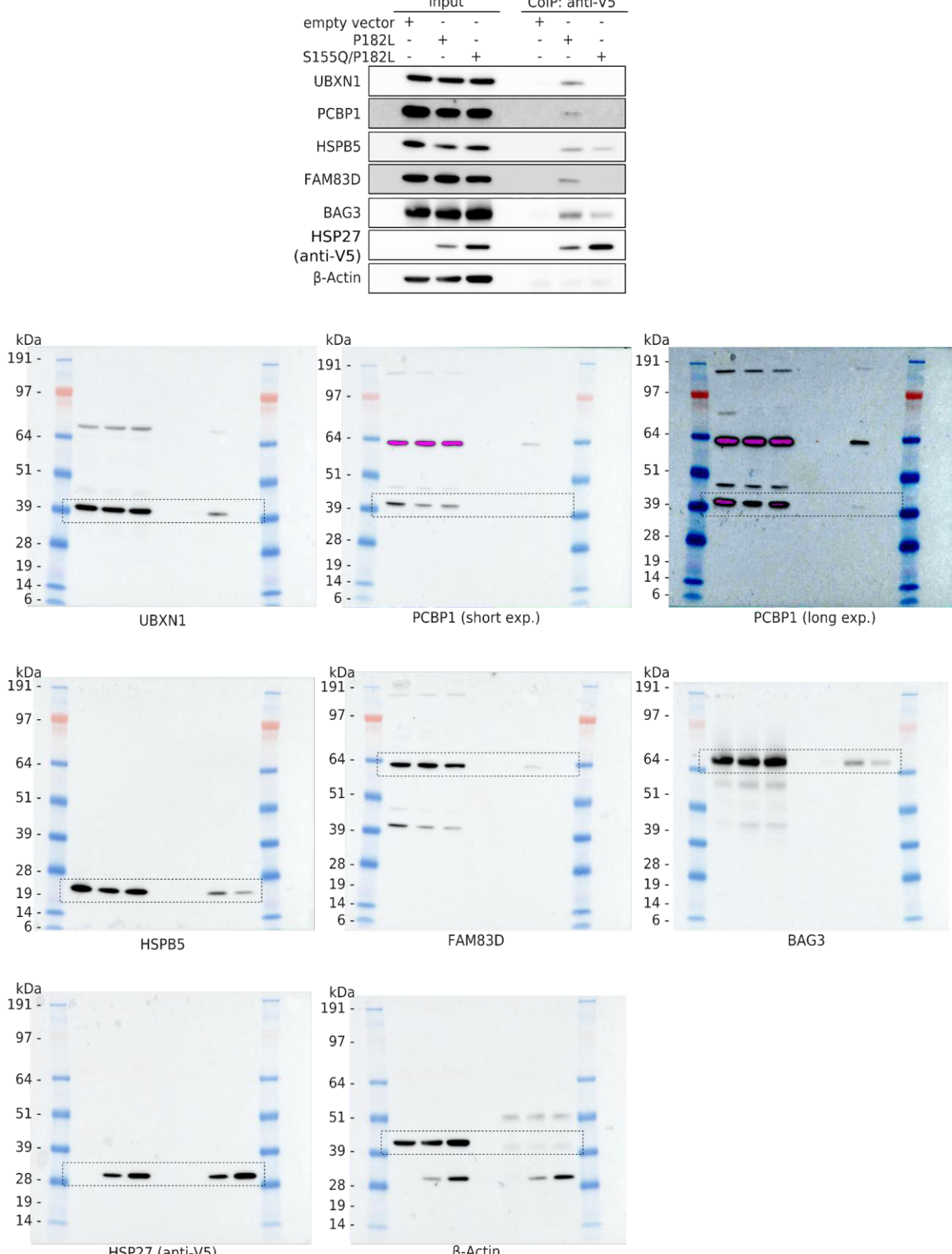

Supplement: Supplementary file 5 — Source Data for Figure 5 [file EMBJ-40-e103811-s005.pdf]
